# Mg-Protoporphyrin IX Signals Enhance Plant’s Tolerance to Cold Stress

**DOI:** 10.3389/fpls.2016.01545

**Published:** 2016-10-18

**Authors:** Zhong-Wei Zhang, Zi-Li Wu, Ling-Yang Feng, Li-Hua Dong, An-Jun Song, Ming Yuan, Yang-Er Chen, Jian Zeng, Guang-Deng Chen, Shu Yuan

**Affiliations:** ^1^College of Resources, Sichuan Agricultural UniversityChengdu, China; ^2^Key Lab of Aromatic Plant Resources Exploitation and Utilization in Sichuan Higher Education, College of Life Science and Food Engineering, Yibin UniversityYibin, China; ^3^College of Life Sciences, Sichuan Agricultural UniversityYa’an, China

**Keywords:** antioxidant enzyme, cold stress, cyanide-resistant respiration, glutathione, Mg-protoporphyrin IX signals

## Abstract

The relationship between Mg-protoporphyrin IX (Mg-Proto IX) signals and plant’s tolerance to cold stress is investigated. *Arabidopsis* seedlings grown for 3 weeks were pretreated with 2 mM glutamate (Glu) and 2 mM MgCl_2_ for 48 h at room temperature to induce Mg-Proto IX accumulation. Then cold stress was performed at 4°C for additional 72 h. Glu + MgCl_2_ pre-treatments alleviated the subsequent cold stress significantly by rising the leaf temperature through inducing Mg-Proto IX signals. The protective role of Glu + MgCl_2_ treatment was greatly compromised in the mutants of Mg-Proto IX synthesis, Mg-Proto IX signaling, and cyanide-resistant respiration. And the enhancement of cold-responsive gene expression was greatly compromised in the mutants of Mg-Proto IX synthesis, Mg-Proto IX signaling and ABA signaling, but not in the mutant of cyanide-resistant respiration. Cold stress promoted cyanide-resistant respiration and leaf total respiration exponentially, which could be further induced by the Glu + MgCl_2_ treatment. Mg-Proto IX signals also activate antioxidant enzymes and increase non-enzymatic antioxidants [glutathione but not ascorbic acid (AsA)] to maintain redox equilibrium during the cold stress.

## Introduction

Magnesium (Mg) is one of the essential macronutrients for plants. Mg is an irreplaceable constituent of the chlorophyll (Chl) and regulates over 300 enzyme activities (especially for ATPases, protein kinases, glutathione synthase, phosphatases, and RNA polymerases), which are involved in ion absorption and transport (da Silva et al., 2014). Exogenous magnesium fertilizers increase crop yields. Mg^2+^ also has important roles in photosynthesis. Proton gradient is formed acorss the thylakoid lumen after the light perception and the electron transport, which results in charge separation and Mg^2+^ flux from lumen to the stroma (Maathuis, 2009).

Mg^2+^ is the cofactor of a large number of important enzymes and plays a key role in the stabilization of DNA and RNA. The most prominent example of enzyme reactions where Mg^2+^ is irreplaceable, are the processes associated with the phosphorylation-dephosphorylation cycle and energy transfer between ATP and ADP (Maathuis, 2009).

Conjugated Mg is the best known for its central position in the Chl molecule where it covalently coordinates with four *N*-atoms of the porphyrin ring. The enzyme of Mg^2+^ insertion into protoporphyrin is the Mg-chelatase (Maathuis, 2009). Among all kinds of Mg porphyrins, Mg-protoporphyrin IX (Mg-Proto IX, a Chl precursor) is a putative signaling molecule that accumulates in chloroplasts under stress conditions and regulates nuclear photosynthetic gene expression negatively (Zhang et al., 2011a,b).

Recent studies showed that Mg-Proto-IX-derived signals strongly regulate plant resistance to environmental stresses (Zhang et al., 2015). Mg-Proto IX signals promote stress-responsive gene expression after herbicide or high-light treatments (Zhang et al., 2011b). Transgenic rice expressing *Myxococcus xanthus* protoporphyrinogen oxidase (PPOX) accumulated Proto IX and Mg-Proto IX and had greater tolerance to drought stress than the wild-type plants (Phung et al., 2011). Rice coproporphyrinogen III oxidase mutant *rlin1* (with less Mg-Proto IX accumulation) showed necrotic lesions on plant leaves (Sun et al., 2011).

*GUN5* encodes the Mg-chelatase H subunit, the enzyme that introduces Magnesium ion into the porphyrin ring as a rate-limiting step of Chl biosynthesis (Strand et al., 2003). Plastid GUN1 (genomes uncoupled protein 1) and nuclear ABI4 (an Apetala 2-type ABA-insensitive transcription factor) are key factors in Mg-Proto IX signaling (Koussevitzky et al., 2007). ABI4 also is a key component of the ABA signaling pathway. Germination suppression sensitivity of *gun4* and *gun5* mutant seeds to ABA was increased compared to the wild-type seeds (Voigt et al., 2010). The *gun1* mutant was more sensitive to ABA than the wild-type plants, implying that GUN1 participates in ABA signaling (Cottage et al., 2010). All of the data suggest that tetrapyrrole-plastid signals and ABA signals may be interconnected.

Mg-Proto-IX-synthesis mutant *gun5* and Mg-Proto-IX-signaling mutants *gun1* and *abi4* had impaired basal high-temperature-tolerance (Miller et al., 2007) and cold-tolerance (Tang et al., 2014). The *gun5*, *gun1* and *abi4* mutants had more oxidative damages than the wild-type plants under water stress (Cheng et al., 2011). The *gun1* mutant had impaired drought-tolerance (Zhang et al., 2013). All of the studies indicated the physiological role of Mg-Proto IX signaling in plant’s stress adaption (Zhang et al., 2015).

The mitochondrial respiratory chain in higher plants consists of the ATP-coupling cytochrome pathway (CP) and the cyanide (CN)-resistant alternative respiratory pathway. The alternative pathway branches from the main respiratory electron transport chain after the site of ubiquinone pool, and bypasses the last few steps of the cytochrome respiratory pathway, where its terminal oxidase is the alternative oxidase (AOX) (Vanlerberghe and McIntosh, 1997). Early studies suggested that the alternative pathway consists in thermogenic tissues of plant, produces heat, and then induces florescence (Vanlerberghe and McIntosh, 1997). Moreover, AOX also scavenges reactive oxygen species (ROS) and enhances plant’s cold-stress tolerance through thermogenesis (Tang et al., 2014).

Under the high-light stress, reducing equivalents (NADPH) from chloroplast are transported to mitochondrion by the malate/oxaloacetate shuttle and then dissipated mainly by the AOX. Thus, AOX plays a key role in chloroplast-mitochondria interactions (Zhang et al., 2016). AOX is encoded by a small nuclear multigene family that comprises at least two different subfamilies: *AOX1* and *AOX2*. In *Arabidopsis*, five genes encoding the two subfamilies of *AOX* have been identified, namely *AOX1a*, *AOX1b*, *AOX1c*, *AOX1d*, and AOX2. *AOX1a* is the major isoform in leaf and 60–70% of CN-resistant respiration is declined in the *Arabidopsis aox1A* mutant (Zhang et al., 2016). Furthermore, *AOX1a* is a plastid-signal-inducible gene, which is positively regulated by the Mg-Proto IX accumulation (Zhang et al., 2011b), also indicating the chloroplast-mitochondria interaction.

In this study, we studied the relationship between plastid Mg-Proto IX signals and the cold stress tolerance with multiple *Arabidopsis* mutants. We found that cold-induced cyanide-resistant respiration plays a dominate role in thermogenesis during the stress condition, which could be further promoted by the Mg-Proto IX signal. Mg-Proto IX signals also enhance antioxidant enzyme activities and accumulate non-enzymatic antioxidants (mainly glutathione) to maintain redox equilibrium during the cold stress.

## Materials and Methods

### Plant Materials and Growth Conditions

*gun5* and *gun1 Arabidopsis* mutant seeds were obtained from Prof. Joanne Chory (Salk Institute, La Jolla, CA, USA) and Dr. Enrique López-Juez (University of London, Egham, UK). Other *Arabidopsis thaliana* mutants were purchased from the *Arabidopsis* Biological Resource Center (Ohio State University, Columbus, OH, USA). *Arabidopsis* seedlings were grown in soil under a 16:8-h light–dark cycle of medium light (100 μ mol m^-2^ s^-1^) at 25°C for 3 weeks.

### Magnesium Chloride, Glutamate, and Cold Treatments

Three-weeks-old *Arabidopsis* seedlings were pretreated (sprayed on the leaves directly) with 2 mM (or 8 mM) Glu, 2 mM magnesium chloride (MgCl_2_), 0.5 mM Propyl gallate (PG, an inhibitor to cyanide-resistant respiration (Xu et al., 2012) or water (for the untreated control) for 48 h (once a day) at the room temperature. After the pretreatments, cold stress was performed at 4 ± 1°C for additional 72 h.

### Chlorophyll and ALA Determination

Chlorophyll contents were determined by using the equations: Chl *a* (mg/g) = [12.21 × A_663_ - 2.81 × A_646_] × Volume (80% acetone; mL)/[1000 × Weight (tissue; g)]. Chl *b* (mg/g) = [20.13 × A_646_ - 5.03 × A_663_] × Volume/(1000 ×Weight). Total Chls (mg/g) = [17.32 × A_646_ + 7.18 × A_663_] ×Volume/(1000 × Weight) (Lichtenthaler and Wellburn, 1983). The amount of formed 5-aminolevulinic acid (ALA) was measured according to the method of Dei (1985).

### Fluorescence HPLC Analysis of Proto IX and Mg-Proto IX

Pigments from 1 g seedlings were extracted with 2 ml acetone: 0.2 M NH_3_⋅H_2_O (9:1, v/v). The extracts were subjected to High Performance Liquid Chromatography (HPLC). 0–10.3 min eluate was detected with an excitation at 417 nm and an emission at 595 nm, and then a change to 402 nm excitation and emission at 633 nm (Mock and Grimm, 1997; Mochizuki et al., 2008). Mixes of 1 pmol Mg-Proto IX and 1 pmol Proto IX were used as authentic standards.

### *Arabidopsis* Leaf Respiration Measurement

A Clark electrode (Hansatech, King’s Lynn, UK) was used. *Arabidopsis* leaves were cut into fine pieces, and then adjusted to approximately 2.5 mg dry weight *per* mL before suspended in working buffer in the oxygen electrode cuvette. 1 mM KCN or 20 μM PG was added to the buffer for CP inhibition and CN-resistant pathway inhibition, respectively. O_2_ uptake value in the presence of KCN but sensitive to PG indicates the CN-resistant respiration capacity. While the O_2_ uptake value without KCN or PG indicates the total respiration (Lei et al., 2008).

### Measurement of CO_2_ Assimilation Rate

An open photosynthesis system TPS-1 (PP systems, Hitchin, UK) was used. CO_2_ assimilation rate (*P*n) was measured at 360 μmol mol^-1^ CO_2_ concentration and illumination of 0–1600 μmol m^-2^ s^-1^ at 25°C (Liu et al., 2009; Chen et al., 2015).

### Determination of Antioxidant Enzymes

For determination of antioxidant enzymes, 0.3 g of *Arabidopsis* leaf were grounded in 3 mL extraction buffer containing 25 mM Hepes, 2% polyvinyl-pyrrolidone (PVP), 0.2 mM EDTA and 2 mM ascorbate (pH 7.8). After 20 min 12,000 × *g* centrifugation at 4°C, the supernatants were collected (Cao et al., 2009). Ascorbate peroxidase (APX), superoxide dismutase (SOD), glutathione reductase (GR), peroxidase (POD), and dehydroascorbate reductase (DHAR) activities were determined (Cao et al., 2009). The SOD assay buffer contains 50 mM phosphate, 12 mM L-methionine, 0.1 mM EDTA, 75 μM nitro blue tetrazolium (NBT) and 2 μM riboflavin (pH 7.8). Reaction condition was 15 min light of 50 μmol m^-2^ s^-1^ with simultaneous shaking. Then the OD_560_ was detected. The POD assay buffer contains 50 mM phosphate, buffer, 40 mM H_2_O_2_ and 10 mM guaiacol (pH 7.0). Then the OD_470_ was detected at 20°C. The APX assay buffer contains 0.1 mM EDTA, 50 mM Hepes-KOH, 0.2 mM H_2_O_2_ and 0.5 mM AsA (pH 7.6). Then the OD_290_ was detected. Reduced ascorbic acid/dehydroascorbate (AsA/DHA) and reduced glutathione/oxidized glutathione (GSH/GSSG) and ratios were determined as indicated previously. GR assay buffer contains 1 mM EDTA, 100 mM Tris-HCl, 0.2 mM NADPH, and1 mM GSSG (pH 8.0). Then the OD_340_ was detected. DHAR assay buffer contains 1 mM EDTA, 100 mM Hepes-KOH, 0.2 mM DHAR, and 2.5 mM GSH (pH 7.0). Then the OD_265_ was detected (Cao et al., 2009; Chen et al., 2015).

### Hydrogen Peroxide and Superoxide Staining and Quantification

Hydrogen peroxide and superoxide in leaves were visualized by 3,3-diaminobenzidine (DAB) and NBT, respectively. *Arabidopsis* leaves were cut at the leaf base and infiltrated in 0.5 mg mL^-1^ NBT or 2 mg mL^-1^DAB solution for 2–8 h. Samples were then decolorized in 95% ethanol at 80°C for 0.5–2 h (Yang et al., 2004).

H_2_O_2_ content was measured as described by Velikova et al. (2000). Approximately 0.5 g leaves were homogenized in ice bath with 5 mL 0.1% (m/v) trichloroacetic acid (TCA). The homogenate was centrifuged at 12,000 × *g* for 20 min at 4°C, 0.5 mL of the supernatant was added to 0.5 mL, 10 mM potassium phosphate buffer (pH 7.0) and 1 mL 1 M KI. The absorbance of supernatant was read at 390 nm.

Superoxide was quantitated by the hydroxylamine method (Elstner and Heupel, 1976). 1 g tissue was homogenized in 65 mM potassium phosphate buffer (3 mL; pH 7.8). The homogenate was centrifuged at (10,000 × *g* for 15 min). The supernatant (0.5 mL) was added to 65 mM potassium phosphate buffer (0.5 mL; pH 7.8) containing 10 mM hydroxylammoniumchloride (0.1 mL) and incubated (25°C for 20 min). Sulphanilic acid (58 mM; 1 mL) and α-naphthyl amine (7 mM; 1 mL) were added to the mixture, and it was allowed to incubate (25°C for 20 min). The final solution was mixed with an equal volume of chloroform and the absorbance of the pink phase was measured at 530 nm.

### RWC and MDA Content Measurements

Relative water content (RWC) is defined as the following equation: RWC = (fresh weight - dry weight)/(turgid weight - dry weight) × 100%.

Leaf malonyldialdehyde (MDA) level was detected as described previously. About 0.2 g *Arabidopsis* leaves were grounded in 5 mL ice-cold 5% TCA. After 10 min 8,000 × *g* centrifugation at 4°C, supernatant was transferred into a new EP tube and then 20% TCA with 0.5% thiobarbituric acid (TBA) was added. The mixture was boiled for 0.5–1 h, and then 10 min 8,000 × *g* centrifugation was performed. OD values (OD_535_ - OD_600_) of the supernatant were measured. The extinction coefficient is 155 mM^-1^ cm^-1^ (Cao et al., 2009).

### Measurement of Leaf Temperature

*Arabidopsis* leaf temperature was visualized by a FLIR T620 thermal-imaging camera (Thermal CAM-FLIR Systems, USA). Individual pot was transferred from the 4°C cabinet to the room temperature. Then within 60 s after the transfer (about 45–60 s), the thermal-photo was taken. The thermal image was analyzed by the software accompanying with the camera. Average leaf temperatures based on the leaf area are shown.

### RNA Isolation, cDNA Synthesis, and qPCR

RNA was isolated simultaneously with the TRIzol RNA kit (Invitrogen, Carlsbad, CA, USA). RNA The purification of RNA samples was detected by measuring the absorbance ratios of A_260_/A_280_, which in all the samples were about 1.9. cDNA was synthesized from RNA templates following the SYBR Premix Ex Taq (TaKaRa, Dalian, China) protocol. The primers used for qPCR are same as in Park et al. (2015). qPCR was performed with SYBR-Green chemistry using the Eppendorf Realplex Mastercycler. The *Ct* (threshold cycle), defined as the PCR cycle at which a statistically significant increase of reporter fluorescence was first detected, was used as a measure for the starting copy numbers of the target gene (Czechowski et al., 2005; Yuan et al., 2016). Three technical replicates were performed for each experiment. *ACTIN1* gene (At2g37620) was used as internal controls.

### Western Blotting to CBF1 Protein

SDS - polyacrylamide gel electrophoresis (SDS-PAGE) and Western blotting analysis of the extracts were processed according to the method as described previously (Zhang et al., 2013; Yuan et al., 2016). Leaf total protein extracts (20 μg) were loaded in each lane. For Western Blotting, the proteins were electron-transferred onto nitrocellulose films. Antibodies used were anti-tumidinoda CBF1 (a gift from Dr. Qibing Chen at Sichuan Agricultural University) and anti-*Arabidopsis* ACTIN1 IgG (AgriSera Comp., Umea, Sweden). Alkaline phosphatase-conjugated antibodies were used as the secondary antibodies.

### Statistics Analysis

Three to five rosette seedlings were collected for each treatment. The typical results are shown as mean values of three biological replicates with standard deviations (±SD). The Student’s *t*-test was performed for all the data. Significant differences were identified with the *p*-value of 0.05.

## Results

### Effects of Glutamate + MgCl_2_ Pre-treatments on Mg-Proto IX Levels and Leaf Temperatures

**FIGURE 1:**
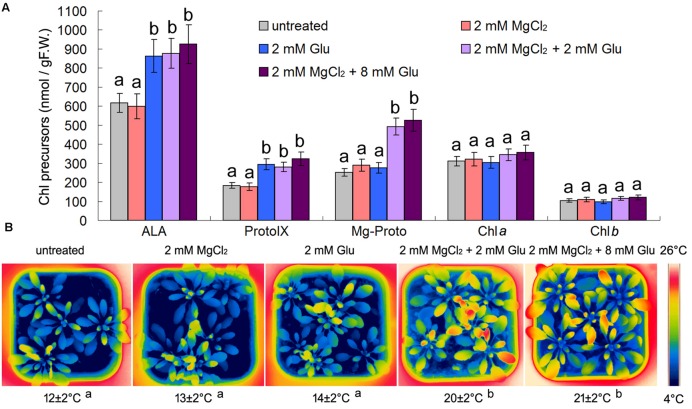
**Effects of 2 or 8 mM glutamate (Glu) + 2 mM MgCl_2_ pre-treatments on Mg-Proto IX and chlorophyll (Chl) synthesis at room temperature (A) and leaf temperature after the subsequent cold stress (B).** After the pretreatments with Glu and MgCl_2_ for 48 h at the room temperature, cold stress was performed at 4 ± 1°C for additional 72 h. Levels of Chl precursors ALA, Proto IX, and Mg-Proto IX in 21-day wild-type *Arabidopsis* seedlings were determined **(A)**. F.W., fresh weight. Error bars show standard deviations (*n* = 3). *Arabidopsis* leaf temperature was visualized by a FLIR T620 thermal-imaging camera. Average leaf temperatures based on the leaf area are shown below corresponding thermal images **(B)**. Multiple comparisons using the least significant difference (LSD) method, uppercase letters represent a significant level of 0.05. With the same letter are not significantly different between the treatments.

Considering the possible relationship between Mg-Proto IX signaling and cold-stress tolerance mentioned above (Tang et al., 2014), we used some chemical pre-treatments to trigger Mg-Proto IX signals before the cold stress onset. One molecule of Mg-Proto IX comes from four molecules of Glu and one molecule of magnesium ion. Therefore, we used 8 mM Glu and 2 mM magnesium chloride to induce Mg-Proto IX accumulation. However, from **Figure 1A**, we know that 8 mM Glu + 2 mM MgCl_2_ and 2 mM Glu + 2 mM MgCl_2_ had a similar effect on Mg-Proto IX synthesis (the difference was not significant, *p* > 0.05). While solo magnesium chloride treatment or solo Glu treatment could not induce Mg-Proto IX accumulation, although solo Glu treatment apparently induced 5-aminolevulinic acid (ALA) accumulation. Therefore, the concentration of 2 mM Glu was selected for further studies.

Mg-Proto IX accumulation did not induce apparent temperature rising under the normal growth condition of the room temperature (increasing extent <3°C; data not shown). However, Glu + MgCl_2_ pre-treatments alleviated the subsequent cold stress significantly by rising the leaf temperature (about 8°C higher than the control). 8 mM Glu + 2 mM MgCl_2_ and 2 mM Glu + 2 mM MgCl_2_ had a similar effect on leaf temperature (the difference was not significant, *p* > 0.05). While solo magnesium chloride treatment or solo Glu treatment could not induce leaf temperature enhancement (**Figure 1B**).

### The Protective Role of Glutamate + MgCl_2_ Treatments on Cold-Stress

**FIGURE 2:**
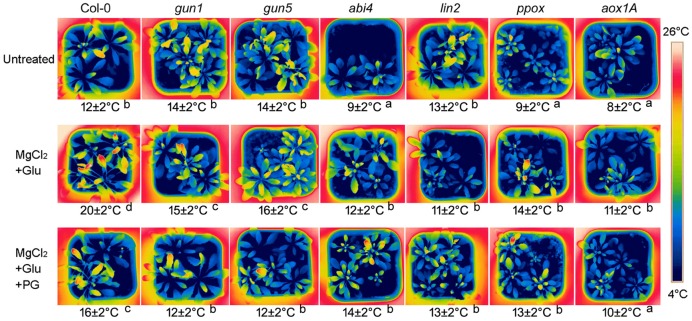
**Effects of 2 mM Glu + 2 mM MgCl_2_ pre-treatments with or without 0.5 mM Propyl gallate (PG) on leaf temperature after the subsequent cold stress.** After the pretreatments with Glu, MgCl_2_, or PG for 48 h at the room temperature, cold stress was performed at 4 ± 1°C for additional 72 h. Then the thermal images were taken immediately. Average leaf temperatures based on the leaf area are shown below corresponding thermal images. Col-0, the wild-type plant. Different lowercase letters indicate significant differences at 0.05 (*P* < 0.05) levels.

Glutamate + MgCl_2_ –induced leaf temperature enhancement was greatly compromised in *Arabidopsis* Mg-chelatase H subunit mutant (*gun5*), protoporphyrinogen oxidase mutant (*ppox*) and coproporphyrinogen III oxidase mutant (*lin2*), all of which accumulate less Mg-Proto IX than the wild-type. Glu + MgCl_2_ treatment also could not prompt leaf temperature of *gun1* and *abi4* mutants, whose Mg-Proto IX signaling is blocked (**Figure 2**).

At 28°C, *Arabidopsis* plants exhibit longer petioles, larger leaves and have faster growth and development compared to those grown at 21 degrees. Glu + MgCl_2_ treatment at 21°C resulted in similar phenotypic effects of 28°C. While the increasing of leaf area and seedling fresh weight was greatly compromised in the mutants of Mg-Proto IX synthesis or Mg-Proto IX signaling (Supplementary Figure S1). These data confirmed the role of Mg-Proto IX in cold stress tolerance.

Our previous study showed that among stress-responsive genes, the AOX gene *AOX1a* was prominently induced by Mg-Proto IX signals (Zhang et al., 2011b). AOX is a key enzyme of CN-resistant respiration, which process produces heat, especially under the cold stress or during the flowering for volatilizing scents (Vanlerberghe and McIntosh, 1997; Seymour and Gibernau, 2008). Therefore, the role of CN-resistant respiration in Mg-Proto-IX-induced leaf temperature enhancement was investigated further. Leaf temperatures were always low in *aox1A* mutant or in the seedlings treated with PG (an inhibitor to CN-resistant respiration), no matter with or without Glu + MgCl_2_ pre-treatments (**Figure 2**), suggesting the dominated role of CN-resistant respiration in Mg-Proto-IX-mediated cold tolerance.

**FIGURE 3:**
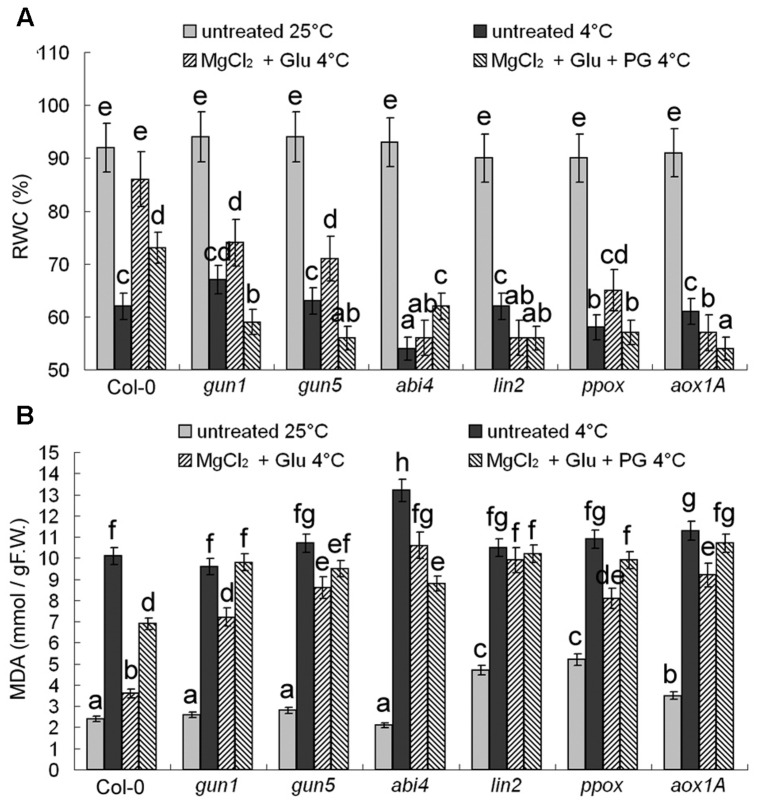
**Effects of 2 mM Glu + 2 mM MgCl_2_ pre-treatments with or without 0.5 mM PG on relative water content (RWC) (A) and malonyldialdehyde (MDA) (B) content after the subsequent cold stress of 4°C for 72 h.** After the pretreatments with Glu, MgCl_2_, or PG for 48 h at the room temperature, cold stress was performed at 4 ± 1°C for additional 72 h. F.W., fresh weight. Error bars show standard deviations (*n* = 3). Different lowercase letters indicate significant differences at 0.05 (*P* < 0.05) levels.

Changes of RWC and MDA content (**Figure 3**) confirmed that the protective role of Glu + MgCl_2_ treatments was greatly compromised in all the mutants of Mg-Proto IX synthesis, Mg-Proto IX signaling, or CN-resistant respiration.

### Mg-Proto IX Signaling Enhances Cold Tolerance by Inducing Cold-Responsive Gene Expression

**FIGURE 4:**
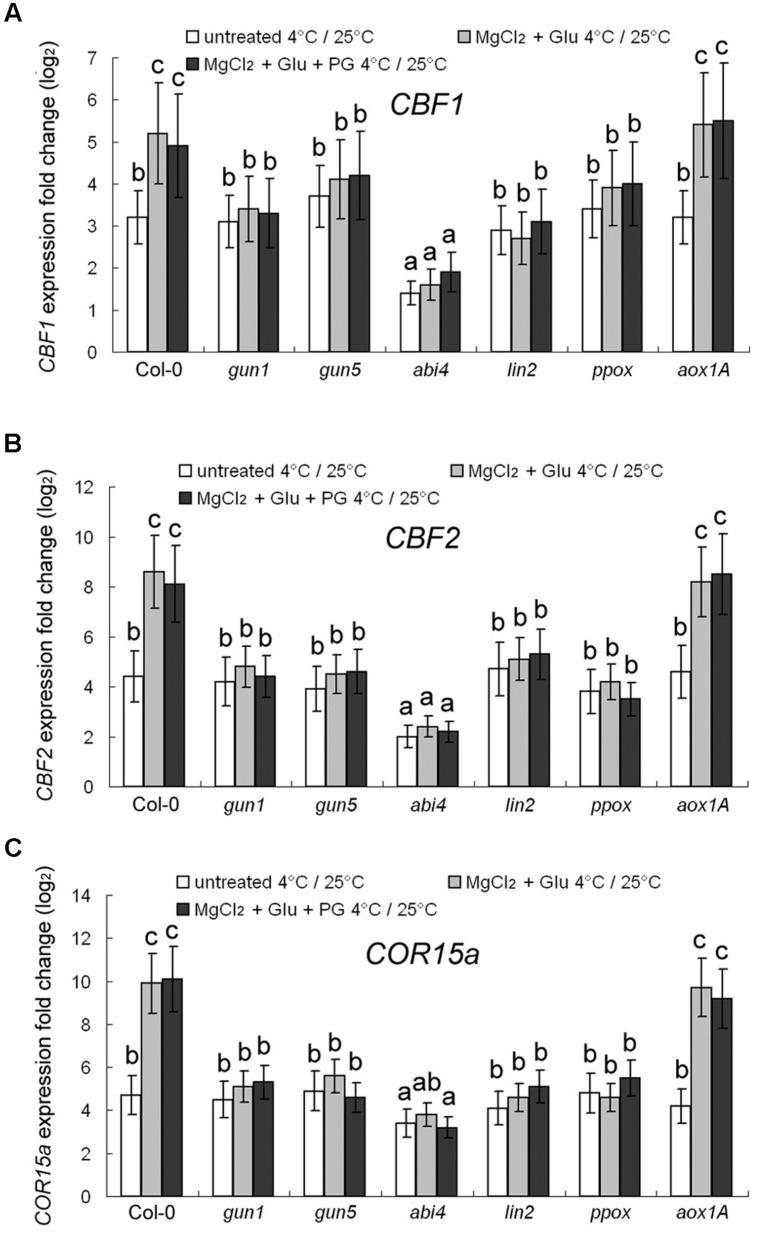
**Transcriptional regulation of *CBF1* (A), *CBF2* (B), and *COR15a* (C) genes by cold and Mg-Proto IX.** After the pretreatments with 2 mM Glu + 2 mM MgCl_2_ or 0.5 mM PG for 48 h at the room temperature, cold stress was performed at 4 ± 1°C for additional 72 h. Transcript expression levels of *CBF1*, *CBF2*, and *COR15a* genes were examined using qPCR. Ct values were normalized to *ACTIN1* controls and are expressed relative to those of the plants grown at the room temperature (without any pretreatment). The average log_2_ fold change after the 25°C to 4°C transfer is shown. Error bars show standard deviations (*n* = 3). Different lowercase letters indicate significant differences at 0.05 (*P* < 0.05) levels.

Expression of three cold-responsive genes was investigated. C-repeat Binding Factors (CBF) 1 and 2 (Park et al., 2015) and Cold-Regulated protein COR15a (a 15-kDa polypeptide targeted to the chloroplast; Steponkus et al., 1998) are cold-responsive marker proteins. 72–h 4°C cold-stress induced 3.2-fold, 4.4-fold and 4.7-fold increasing of *CBF1*, *CBF2*, and *COR15a* transcripts, respectively, compared to the control seedlings grown at 25°C (**Figure 4**). However, the cold-induced gene-expression enhancement was largely compromised in *abi4* mutant (*ABA Insensitive 4*), but not in other mutants, implying that ABA signaling may also participate in cold-stress responses. The cold-responsive gene expression could be further promoted by the Glu + MgCl_2_ treatment. However, this effect has not been observed for all the mutants of Mg-Proto IX synthesis or Mg-Proto IX signaling (**Figure 4**), also indicating the key role of Mg-Proto-IX signaling in cold-stress tolerance. PG treatments barely affected cold-responsive gene expression (**Figure 4**).

To be more convincing, western blot analysis to CBF1 protein was performed. Changes at the translational level were consistent with those at the transcriptional level (Supplementary Figure S2), which excluded the secondary co-regulatory effects of cold stress at the post-transcriptional level.

### Effects of Cold Stress and Glutamate + MgCl_2_ Treatments on Chlorophyll Synthesis

**FIGURE 5:**
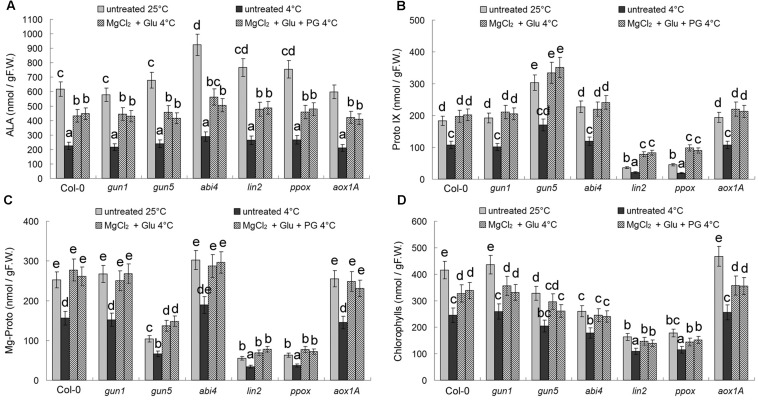
**Effects of 2 mM Glu + 2 mM MgCl_2_ pre-treatments with or without 0.5 mM PG on Chls and their three precursors after the subsequent cold stress.** After the pretreatments with Glu, MgCl_2_, or PG for 48 h at the room temperature, cold stress was performed at 4 ± 1°C for additional 72 h. Levels of Chl precursors ALA **(A)**, Proto IX **(B)**, Mg-Proto IX **(C)**, and seedling total Chls **(D)** were determined. F.W., fresh weight. Error bars show standard deviations (*n* = 3). Different lowercase letters indicate significant differences at 0.05 (*P* < 0.05) levels.

Cold stress led to suppression of Chl synthesis. Chls and all Chl precursors (ALA, Proto IX, and Mg-Proto IX) decreased obviously after the cold stress (**Figure 5**). Glu + MgCl_2_ treatments induced Mg-Proto IX accumulation significantly, as well as ALA, Proto IX and Chls, therefore offsetting the effect of cold stress on Chl synthesis. Because of the mutations in Chl biosynthesis enzymes, Proto IX levels were greatly reduced in *lin2* and *ppox* mutants, and Mg-Proto IX levels were greatly reduced in *gun5*, *lin2*, and *ppox* mutants. As mentioned above, PG is a CN-resistant respiration specific inhibitor, and thus did not affect Chl levels or the precursor levels (**Figure 5**).

### Effects of Cold Stress and Glutamate + MgCl_2_ Treatments on Carbon Assimilation and Dissimilation

**FIGURE 6:**
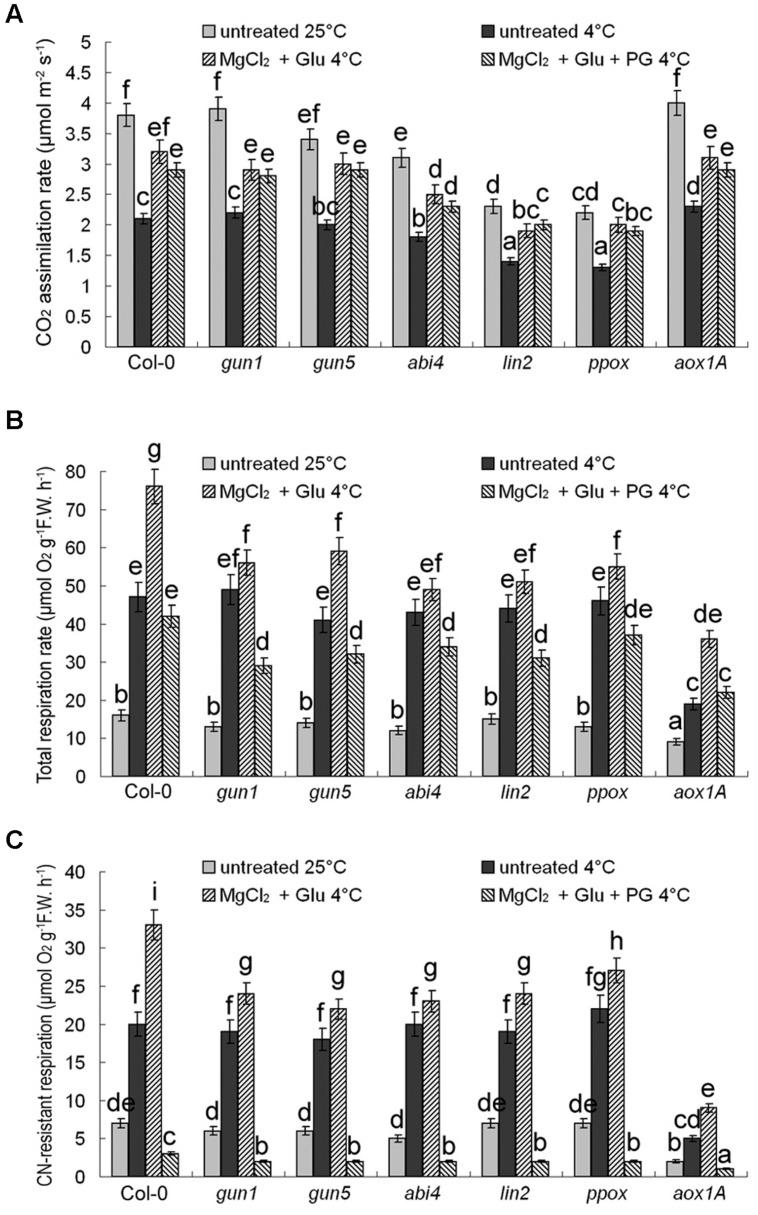
**Effects of 2 mM Glu + 2 mM MgCl_2_ pre-treatments with or without 0.5 mM PG on CO_2_ assimilation rate (A), CN-resistant respiration rate (B), and leaf total respiration rate (C) after the subsequent cold stress.** After the pretreatments with Glu, MgCl_2_, or PG for 48 h at the room temperature, cold stress was performed at 4 ± 1°C for additional 72 h. F.W., fresh weight. Error bars show standard deviations (*n* = 3). Different lowercase letters indicate significant differences at 0.05 (*P* < 0.05) levels.

CO_2_ assimilation rates were positively related with their Chl levels (decreased after cold stress, but increased by the Glu + MgCl_2_ treatment; **Figure 6A**). On the contrary, cold stress promoted CN-resistant respiration and total respiration exponentially, which could be further induced by the Glu + MgCl_2_ treatment. While PG or mutation in *AOX1a* gene effectively inhibited CN-resistant respiration (**Figures 6B,C**). Induction of CN-resistant respiration by the Glu + MgCl_2_ treatment was greatly compromised in all the mutants of Mg-Proto IX synthesis and Mg-Proto IX signaling (**Figures 6B,C**), indicating the role of Mg-Proto IX signaling in CN-resistant respiration enhancement.

### Effects of Cold Stress and Glutamate + MgCl_2_ Treatments on Reactive Oxidative Species Metabolism

**FIGURE 7:**
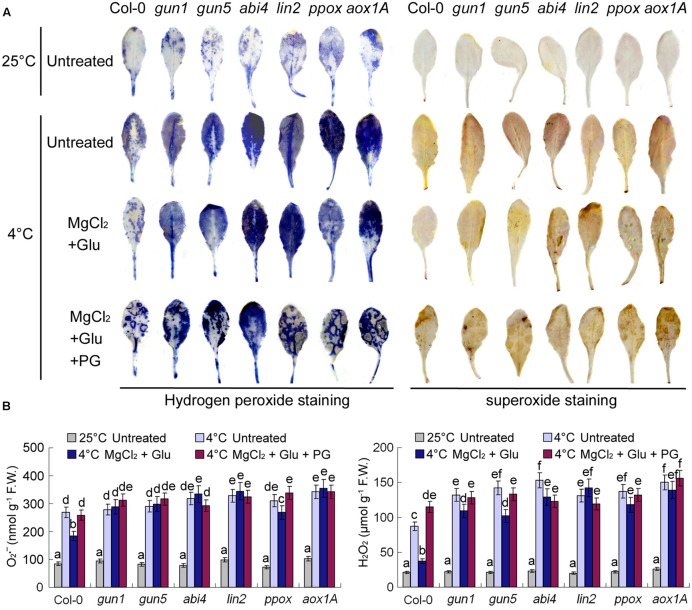
**Superoxide and H_2_O_2_ staining of leaves pre-treated with Glu, MgCl_2_ with or without 0.5 mM PG and after the subsequent cold stress.** After the pretreatments with Glu, MgCl_2_, or PG for 48 h at the room temperature, cold stress was performed at 4 ± 1°C for additional 72 h. Hydrogen peroxide and superoxide in leaves were visualized by DAB and NBT, respectively **(A)**. Quantitative values for all the treatments are shown on the lower panel **(B)**. F.W., fresh weight. Error bars show standard deviations (*n* = 3). Different lowercase letters indicate significant differences at 0.05 (*P* < 0.05) levels.

Superoxide and hydrogen peroxide (H_2_O_2_) are two major types of reactive oxidative species (ROS) in plant cells. Consistent with RWC and MDA contents indicated in **Figure 3**, cold stress increased cellular ROS levels dramatically, while Glu + MgCl_2_ treatments offset the effect of cold stress on ROS accumulation (**Figure 7**). Both superoxide and hydrogen peroxide levels were significantly higher in all the mutants than those in the wild-type leaves no matter with or without Glu + MgCl_2_ pre-treatments (**Figure 7**). PG-treated seedlings also accumulated high levels of ROS, indicating the adverse role of PG on plant’s cold tolerance.

**FIGURE 8:**
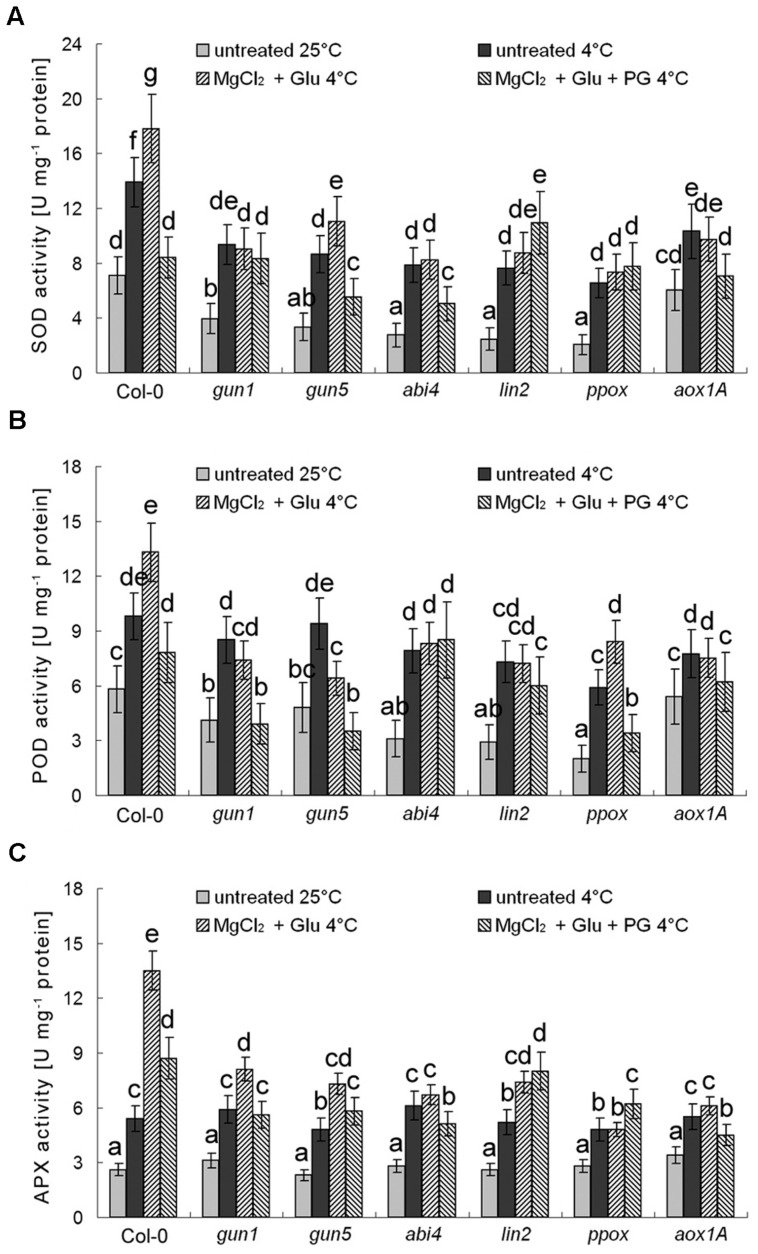
**Effects of 2 mM Glu + 2 mM MgCl_2_ pre-treatments with or without 0.5 mM PG on SOD (A), POD (B), and APX (C) activities after the subsequent cold stress.** After the pretreatments with Glu, MgCl_2_, or PG for 48 h at the room temperature, cold stress was performed at 4 ± 1°C for additional 72 h. Error bars show standard deviations (*n* = 3). Different lowercase letters indicate significant differences at 0.05 (*P* < 0.05) levels.

Besides AOX genes, multiple antioxidant enzyme genes are also Mg-ProtoIX-inducible genes (Strand et al., 2003; Zhang et al., 2011b). As shown in **Figure 8**, POD, SOD, and APX, all these antioxidant enzyme activities were enhanced by the cold stress, which could be further induced by the Glu + MgCl_2_ treatment. While these inductions were greatly compromised in all the mutants or the PG treatment (**Figure 8**).

**FIGURE 9:**
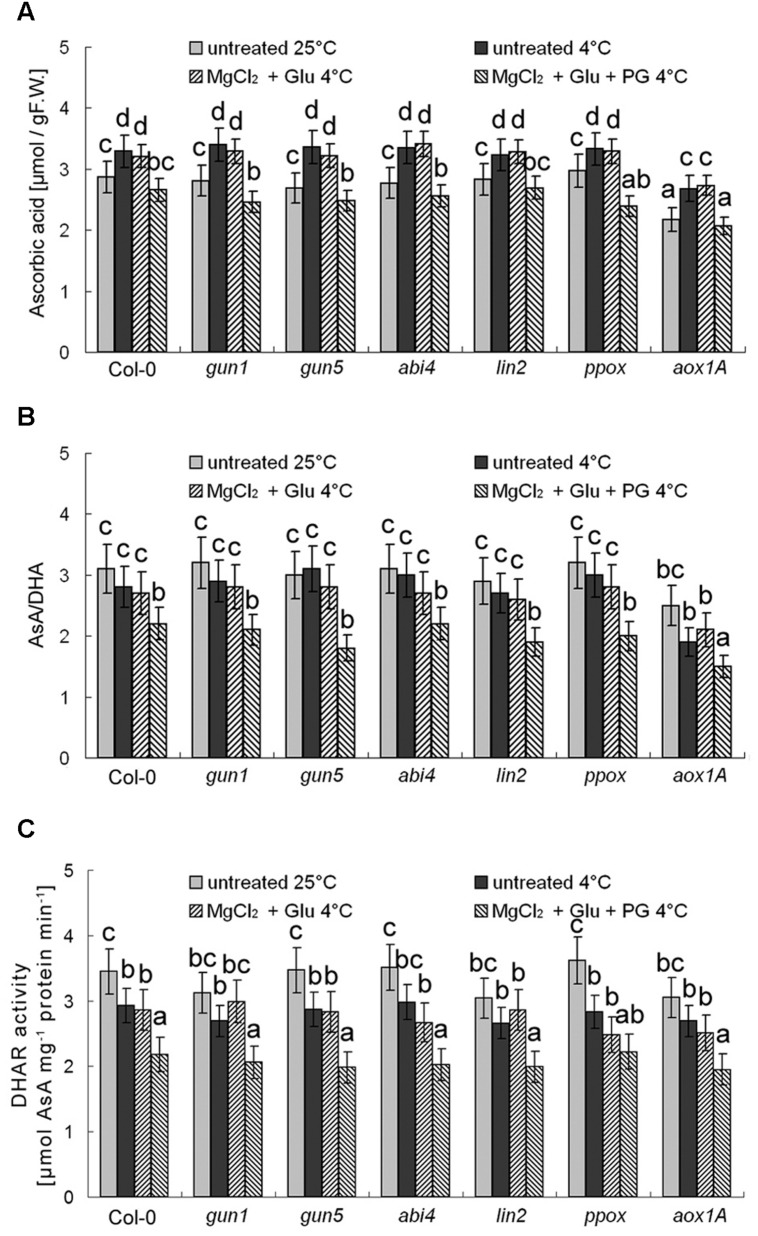
**Effects of 2 mM Glu + 2 mM MgCl_2_ pre-treatments with or without 0.5 mM PG on total AsA level (A), AsA/DHA ratio (B), and DHAR activity (C) after the subsequent cold stress.** After the pretreatments with Glu, MgCl_2_, or PG for 48 h at the room temperature, cold stress was performed at 4 ± 1°C for additional 72 h. F.W., fresh weight. Error bars show standard deviations (*n* = 3). Different lowercase letters indicate significant differences at 0.05 (*P* < 0.05) levels.

**FIGURE 10:**
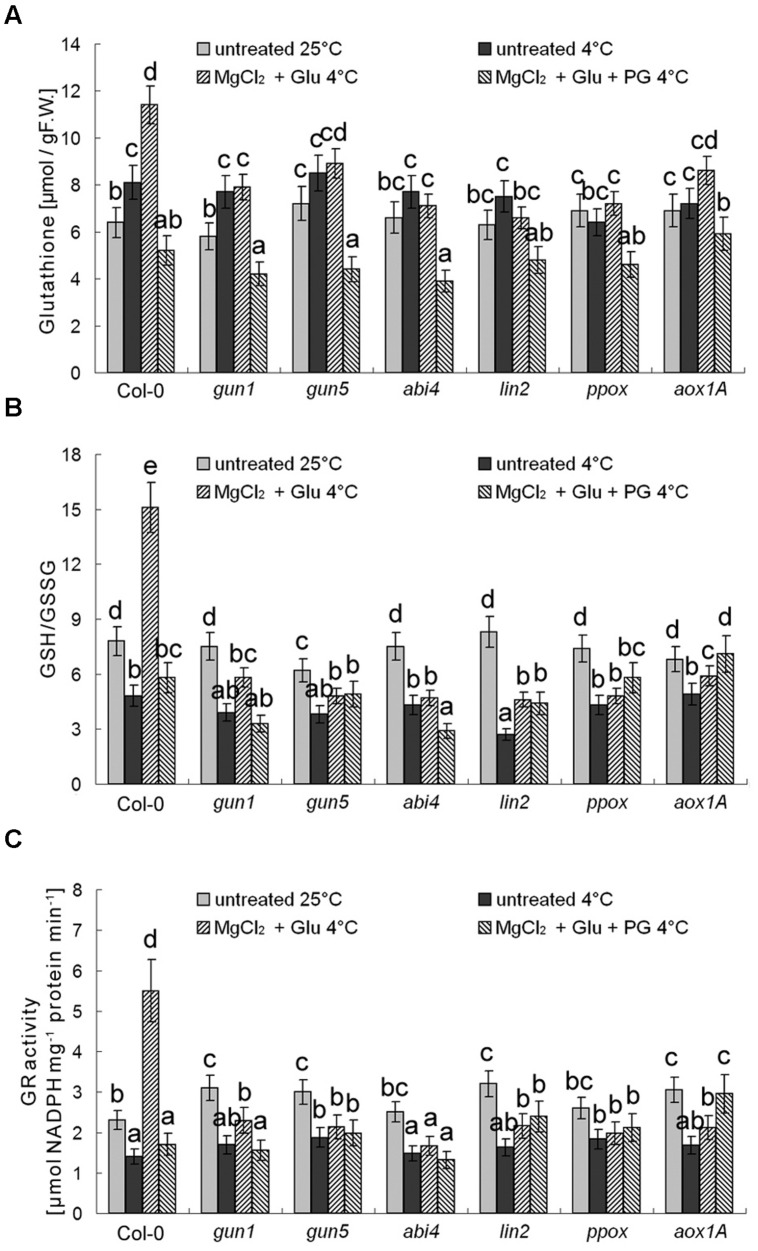
**Effects of 2 mM Glu + 2 mM MgCl_2_ pre-treatments with or without 0.5 mM PG on total GSH level (A), GSH/GSSG ratio (B), and GR activity (C) after the subsequent cold stress.** After the pretreatments with Glu, MgCl_2_, or PG for 48 h at the room temperature, cold stress was performed at 4 ± 1°C for additional 72 h. F.W., fresh weight. Error bars show standard deviations (*n* = 3). Different lowercase letters indicate significant differences at 0.05 (*P* < 0.05) levels.

Besides antioxidant enzymes, there are several non-enzymatic antioxidants important for redox equilibrium, such as AsA and glutathione (GSH; Cao et al., 2009). Interestingly, we found that neither AsA levels nor AsA/DHA (reduced ascorbic acid/dehydroascorbate) ratios (and DHAR activity) were affected by cold stress or Glu + MgCl_2_ treatments (*p* > 0.05; **Figure 9**). Contrastingly, GSH level was increased and GSH/GSSG (reduced glutathione/oxidized glutathione) ratio and GR activity were significantly decreased under the cold stress, both of which could be apparently induced by the Glu + MgCl_2_ treatment (**Figure 10**). These inductions by the Glu + MgCl_2_ treatments were greatly compromised in all the mutants or after the PG treatment (**Figure 10**), suggesting a possible relationship between Mg-Proto IX signaling and glutathione metabolism.

## Discussion

Among all the mutants used in this study, *abi4* mutant had the lowest transcription levels of cold-responsive genes (**Figure 4**). Leaf temperature is highly dependent on stomata opening (by influencing transpiration) and ABA signaling (Costa et al., 2013; Schymanski et al., 2013). Contrastingly, *CBF1*, *CBF2*, and *COR15a* genes showed normal responses to the cold treatment in all the mutants of either Mg-Proto IX synthesis or Mg-Proto IX signaling (**Figure 4**). The results suggest that ABA signaling may be required for basic cold-response, which might be independent of Mg-Proto IX accumulation. However, Mg-Proto IX signals further promote cold-responsive gene expression and thus enhance cold tolerance (Supplementary Figure S3). *CBF* genes encode closely related members of the AP2/ERF family of transcription factors that recognize the C-repeat (CRT)/dehydration-responsive element (DRE) present in the promoters of CBF-targeted genes (Stockinger et al., 1997; Liu et al., 1998; Zwack et al., 2016). Constitutive over-expression of either *CBF1*, *CBF2*, or *CBF3* in transgenic plants leads to altered expression of about a hundred cold-regulated genes (Maruyama et al., 2004; Vogel et al., 2005), and results in an increase in cold tolerance (Liu et al., 1998; Gilmour et al., 2004). The crosstalk between ABA signaling and Mg-Proto IX signaling in the low-temperature regulatory network needs further investigations.

There are two ubiquinol-oxidizing pathways of the respiratory chain in higher plant mitochondria. One is the CP, which is common in the respiratory chain in all aerobic organisms, and the other is the CN-insensitive pathway, which is charged by the AOX. The CN-insensitive pathway branches from the main pathway after the ubiquinone and couples the ubiquinol oxidation and oxygen reduction to form water (Vanlerberghe and McIntosh, 1997). Electron transport during the oxidative phosphorylation results in a proton gradient, which drives ATP production. At the normal condition, cytochrome c oxidase is the terminal electron acceptor, and three ATPs are formed *per* oxygen molecule consumed (Roberts et al., 1984). Contrastingly, for the CN-insensitive pathway, only one ATP is formed *per* oxygen molecule consumed and the extra energy is released as heat (Moore and Siedow, 1991).

Thermogenic plants, such as those belonging to Araceae family, have an ability to maintain constant temperature under cold conditions (Meeuse, 1975; Watling et al., 2006). Actually, thermogenesis through the plant mitochondrial respiratory chain is significant for all plants (Watling et al., 2006; Tang et al., 2014). Our previous study showed that *AOX1a* gene expression was prominently induced by Mg-Proto IX signals (Zhang et al., 2011b). And then induction in CN-resistant respiration greatly enhanced plant’s tolerance to the cold stress (Zhang et al., 2011b). These connections between Mg-Proto IX signals and carbon dissimilation under cold stress conditions have been further proved here by using *Arabidopsis* mutants. *gun1*, *gun5*, *abi4*, *lin2*, and *ppox* mutants developed severe hypothermia as same as the *aox1A* mutant, no matter with or without Glu + MgCl_2_ pre-treatments (to induce Mg-Proto IX signals presumably). Contribution ratio of CN-resistant respiration to the total respiration usually ranges from 20 to 50%. In other words, almost a half of total C dissimilation is regulated by Mg-Proto IX signals, which is important for plant thermogenesis during the cold exposure (Supplementary Figure S3).

Another mechanism of Mg-Proto-IX-mediated cold tolerance is the activation of enzymatic and non-enzymatic antioxidant systems. Previous studies defined some Mg-Proto-IX-signal-inducible genes, including many genes related with oxidative stress, such as *AOX1a*, *POD*, *SOD*, *APX*, *CAT* (encoding Catalase), *FH3* (encoding flavanone 3-hydroxylase), *CHS* (encoding chalcone synthase; Strand et al., 2003; Zhang et al., 2011b). Among non-enzymatic antioxidants, only GSH system (but not AsA system) was found here to be correlated with Mg-Proto IX-signal-enhanced cold stress tolerance.

GSH is a tripeptide with a gamma peptide linkage between the carboxyl group of the Glu side-chain and the amine group of cysteine (Cys), which is a Sulfur (thiol groups)-containing amino acid. Taken up from the soil, sulfate is incorporated into adenosine-5′-phosphosulfate followed by reduction into sulfite and then sulfide and Cys biosynthesis. In parallel, adenosine-5′-phosphosulfate can be further phosphorylated to 3′-phosphoadenosine-5′-phosphosulfate, which is used for sulfation reactions (Mugford et al., 2011). Cys is the key metabolite in the synthesis of sulfur-containing compounds in plants. While the major pool of sulfur is not stored in proteins but is the Cys-containing peptide GSH (Gigolashvili and Kopriva, 2014). GSH is a universal molecule, which plays a crucial role in plants including cellular defense, redox status balance, signal transduction and detoxification (Noctor et al., 2012; Sobrino-Plata et al., 2014). Recently, GSH was shown to modulate the methylglyoxal detoxification systems during high temperature stress (Nahar et al., 2015). Here we show an explicit relationship between GSH (Sulfur metabolism) and Mg-Proto IX signals during cold stress adaptation. Although the detailed biochemical links and signal connections need further investigations.

## Summary

Mg-Proto IX signals (triggered by Glu + MgCl_2_ treatments) enhance plant’s tolerance to cold stress. Both Mg-Proto IX synthesis and signaling are required for the cold tolerance. Mg-Proto IX signals prompt cold-responsive gene expression. Mg-Proto-IX-signal-induced CN-resistant respiration plays a key role in heat production during the cold stress. And the reduced glutathione is also involved in Mg-Proto-IX-signal-mediated cold tolerance.

## Author Contributions

Z-WZ contributed all reagents and materials used in the experiments and wrote the paper. SY designed the experiments and edited the manuscript. Z-LW, L-YF, L-HD, and A-JS performed the experiments. MY, Y-EC, JZ, and G-DC analyzed the data.

## Conflict of Interest Statement

The authors declare that the research was conducted in the absence of any commercial or financial relationships that could be construed as a potential conflict of interest.
