# Supplementary material for: Mg-Protoporphyrin IX Signals Enhance Plant’s Tolerance to Cold Stress
Source: Front Plant Sci. 2016 Oct 18;7:1545. doi: 10.3389/fpls.2016.01545 (PMC5068135; doi:10.3389/fpls.2016.01545)
Supplement: Supplementary file 1 [file Data_Sheet_1.PDF]

A

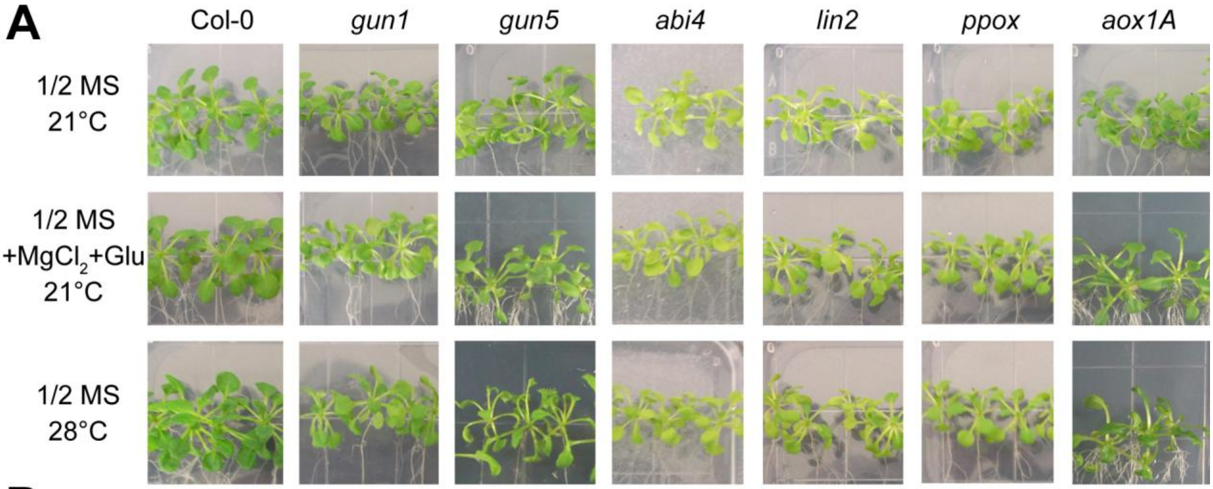

B

Leaf area (mm^2)

	Col-0	*gun1*	*gun5*	*abi4*	*lin2*	*ppox*	*aox1A*
Untreated 21°C	9 ± 2^{a}	9 ± 2^{a}	8 ± 2^{a}	9 ± 2^{a}	7 ± 2^{a}	8 ± 2^{a}	9 ± 2^{a}
$MgCl_2$+Glu 21°C	19 ± 5^{b}	10 ± 2^{a}	11 ± 3^{b}	10 ± 2^{a}	10 ± 2^{b}	11 ± 5^{b}	15 ± 4^{b}
Untreated 28°C	18 ± 4^{b}	12 ± 3^{b}	12 ± 3^{b}	10 ± 3^{a}	11 ± 3^{b}	11 ± 4^{b}	16 ± 4^{b}

C

Fresh weight (g)

	Col-0	*gun1*	*gun5*	*abi4*	*lin2*	*ppox*	*aox1A*
Untreated 21°C	0.26 ± 0.08^{a}	0.28 ± 0.07^{a}	0.24 ± 0.06^{a}	0.24 ± 0.07^{a}	0.20 ± 0.05^{a}	0.21 ± 0.06^{a}	0.28 ± 0.06^{a}
$MgCl_2$+Glu 21°C	0.55 ± 0.14^{b}	0.29 ± 0.08^{a}	0.32 ± 0.09^{b}	0.27 ± 0.09^{a}	0.27 ± 0.08^{b}	0.28 ± 0.11^{b}	0.49 ± 0.11^{b}
Untreated 28°C	0.51 ± 0.15^{b}	0.37 ± 0.08^{b}	0.35 ± 0.09^{b}	0.26 ± 0.08^{a}	0.31 ± 0.09^{b}	0.33 ± 0.09^{b}	0.50 ± 0.10^{b}

FIGURE S1 | Effects of 2 mM glutamate (Glu) + 2 mM $MgCl_2$ co-treatment on seedling development grown on agar plates. Wild-type Arabidopsis seedlings (Col-0) or mutants grew on the 1/2 MS medium at 21 ℃ (with or without Glu + $MgCl_2$ treatment) or 28 ℃ (without Glu + $MgCl_2$ treatment) for 21 days **(A)**. Then the leaf area **(B)** and the seedling fresh weight **(C)** were measured. Multiple comparisons using the least significant difference (LSD) method, uppercase letters represent a significant level of 0.05. With the same letter are not significantly different between the treatments.

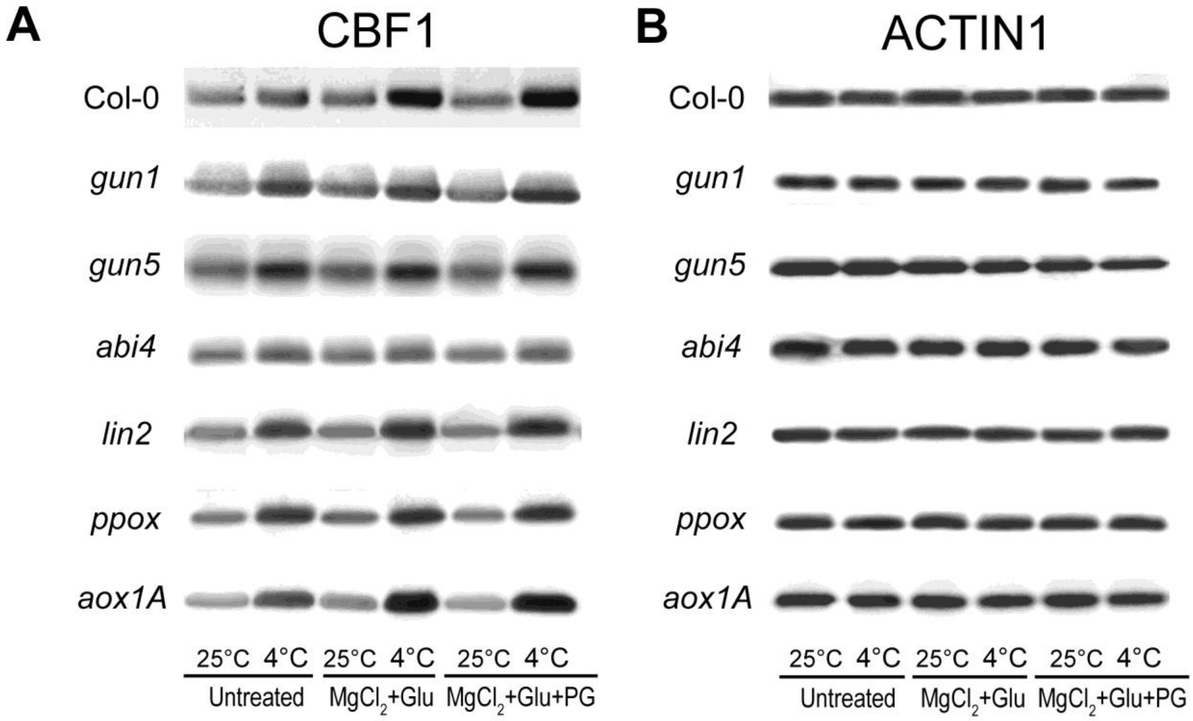

FIGURE S2 | Translational regulation of CBF1 protein by cold and Mg-Proto IX. After the pretreatments with 2 mM glutamate (Glu) + 2 mM $MgCl_2$ or 0.5 mM Propyl gallate (PG) for 48 h at the room temperature, cold stress was performed at 4 ± 1 ℃ for additional 72 h. Translational levels of CBF1 protein were examined by Western blotting **(A)**. ACTIN1 was used as a loading control **(B)**. 72-h 4 ℃ cold-stress induced 2 to 3-fold increasing of CBF1 protein compared to the control seedlings grown at 25 ℃. However, the cold-induced CBF1 enhancement was largely compromised in *abi4* mutant, but not in other mutants. CBF1 protein level could be further promoted by the Glutamate + $MgCl_2$ treatment. However this effect has not been observed for all the mutants of Mg-Proto IX synthesis or Mg-Proto IX signaling. PG treatments barely affected CBF1 protein content.

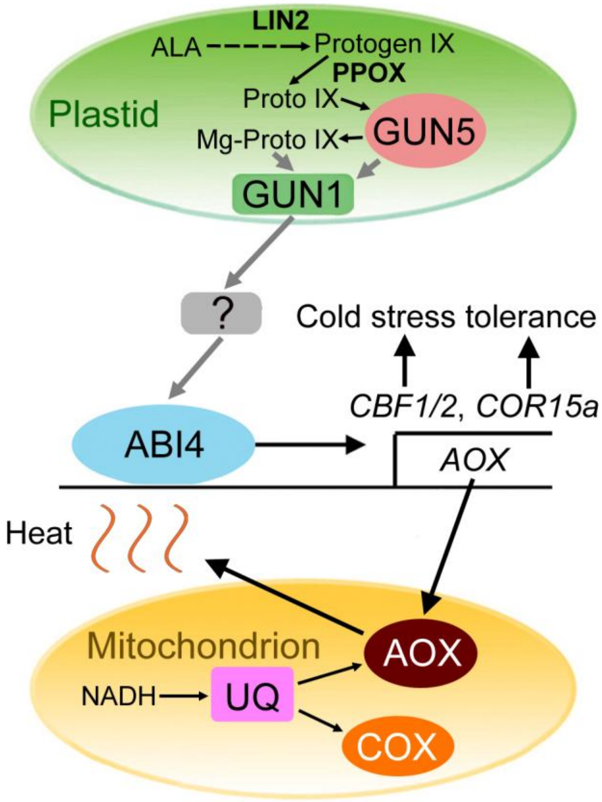

FIGURE S3 | Diagram of Mg-Proto IX-signal and CN-resistant-respiration-regulated cold tolerance. Mg-Proto IX signals (though GUN5-dependent or GUN5-independent pathways) prompt cold-responsive gene expression, such as *CBF1*, *CBF2*, *COR15a* and *AOX*. Plastid GUN1 protein and nuclear ABI4 protein mediate this induction. There are two ubiquinol (UQ)-oxidizing pathways of the respiratory chain in higher plant mitochondria. One is the cytochrome pathway, which is charged by the cytochrome c oxidase COX, and the other is the CN-insensitive pathway, which is charged by the alternative oxidase AOX. CN-resistant respiration plays a key role in heat production during the cold stress.
